# Finite Element Analysis of Crack Propagation in Adhesive Joints with Notched Adherends

**DOI:** 10.3390/ma16010391

**Published:** 2022-12-31

**Authors:** Ayman Qureshi, Tianyue Guan, Marco Alfano

**Affiliations:** Department of Mechanical and Mechatronics Engineering, University of Waterloo, 200 University Avenue West, Waterloo, ON N2L 3G1, Canada

**Keywords:** adhesive joints, DCB, cohesive zone model, crack growth, toughening, snap-through

## Abstract

The adherends notching technique has been the subject of a few recent studies and consists of tailoring the geometry of the adjoined layers to mitigate the bondline peak stresses and enhance the joint strength. In the present study, we explored the effect of the adherends notching technique on crack propagation using finite element (FE) simulations based on the cohesive zone model (CZM) of fracture. Double cantilever beam (DCB) adhesive joints subjected to quasistatic loading were considered as a model material system. An array of equally spaced notches was placed on the faying sides of the adherends, oriented perpendicularly to the direction of crack growth. A parametric investigation was carried out to ascertain the role of the notches and the input cohesive properties on various performance metrics, e.g., load–displacement response and dissipated energy. The proposed notching strategy promotes an unstable crack pinning/depinning process, which effectively delays crack growth and increases the effective work of fracture. Additionally, we found that the overall behaviour is tunable by changing geometric (i.e., notch spacing and depth) and bondline material properties.

## 1. Introduction

Global trends to reduce greenhouse emissions have ignited an interest in lightweight materials, which are poised for significant market growth in the automotive industry. Fuelled by the increasing material mix, which includes aluminum alloys and fibre-reinforced polymers, the search for joining technologies suitable for multimaterial structures is under the spotlight [1]. Recent developments suggest that adhesive bonding can replace traditional fastening methods and may dramatically decrease assembly time and costs [2]. However, because manufacturing and service defects can lead to catastrophic failures, there is still a lack of confidence in solely using adhesives in primary structural applications.

Methods and procedures that can improve the strength and fracture toughness of adhesive joints usually target either the interfaces or the joints’ bondline. For example, several studies have focused on surface treatments, such as plasma [3], laser ablation [4,5], and chemical etching [6], and on the enhancement in bondline properties through the addition of reinforcing particles [7], fibres [8,9], or architected mats [10]. However, strategies that target the geometrical attributes of the adjoined layers to control joint strength and bond toughness are now gaining in popularity [11,12]. This is an emerging topical area that is being further promoted owing to the recent developments in the field of additive manufacturing [13,14].

**Figure 1:**
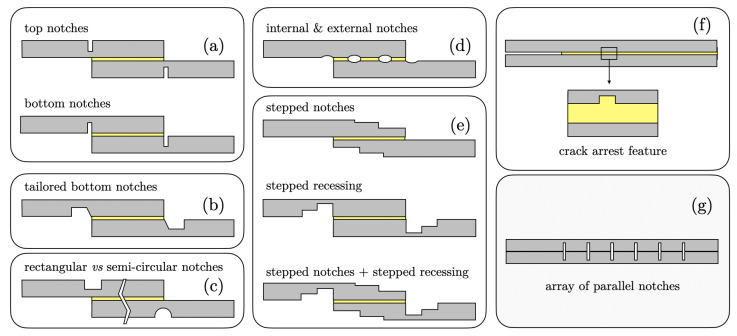
Schematics of adhesive joints with notched adherends. (**a**–**f**) Solutions proposed in previous related studies [15,16,17,18,19,20,21]; (**g**) adhesive-bonded DCB with notched adherends analysed in the present study.

Although dated back to the early 2000s, the adherends notching technique may be listed among these methods [15,16,17,18,19,20,21]. In particular, notches of various geometries (e.g., rectangular or semicircular) are thoughtfully placed in the adherends to reduce bondline stress peaks and increase the joint strength. To the best of our knowledge, the first investigation on the effect of adherend notching on the strength of single lap joints (SLJs) was carried out by Sankatar and Simmons [15]. Two configurations were selected, comprising top notches located at the unbonded surfaces of the adherends and bottom notches located next to the bondline region (see Figure 1a). The authors determined the optimum notch locations and dimensions using finite element analysis. Experimental tests showed a 29% increase in joint strength, which matched a similar decrease in the FE peak peel stress. However, the improvement occurred only for joints featuring a brittle adhesive and ‘top notches’, and the discrepancy between experimental and computational results was attributed to potential nonlinear effects that were not captured by the FE simulations.

In a series of papers, Da Silva and co-workers have recently re-examined the role of adherend notching [16,17,18]. For instance, Bahrami et al. [16] proposed the notching technique to improve the strength of single lap joints and employed an extensive set of FE simulations to tailor the notch shape and location (see Figure 1b). The tailored notch geometry provided a significant improvement in the load-bearing capacity. In a subsequent study, the authors emphasised the interplay between the improved performance and notch depth as well as adhesive properties [17]. On the one hand, the average failure loads of SLJs bonded with a brittle adhesive and a notch depth ratio (ratio of the notch depth to the adherend thickness) of 20% was improved by about 100%. On the other hand, with a ductile adhesive and the same notch depth ratio, the improvement was only ≈25%. Therefore, the finding that the notching technique is more efficient with brittle adhesives was reaffirmed. In [18], the authors extended their study by introducing rectangular and semicircular notches at the outer surface of the adherends (Figure 1c). Interestingly, no significant differences were reported in the obtained joint strength.

Kanani et al. [19] studied SLJs featuring semicircular notches similar to those reported in [18] and which are schematically shown in Figure 1d. However, the notches were simultaneously placed outside and inside the bonding area; to determine an optimal notch shape and location, the authors employed FE-based stress analyses, while the load-bearing capability of the joint was determined using a cohesive zone model (CZM) approach. The results indicated that the notches were able to split the bondline into smaller sections enabling the load to be spread more efficiently, i.e., smaller peak stresses at the joint edges. In comparison with the baseline joint, the strength and maximum failure load of SLJ were significantly improved.

Hacısalihoglu and Akpinar [20] proposed the use of stepped notches, stepped recessing, and a combination of both, as shown in Figure 1e. The authors reported that two- or three-stepped notches allowed for increasing the joint strength by approximately 37% to 38% with respect to the baseline. Stepped notches could reduce the moment caused by eccentric loading; if used in conjunction with the stepped recessing, the strength of the joint increased by more than 50%.

Previous studies focused on the single lap configuration, i.e., a geometry of great practical importance to understand the strength of adhesive joints. However, in a recent study, Hirakawa et al. [21] adopted the double cantilever beam (DCB) configuration and revealed a strong interplay between adherends notching and crack propagation. More specifically, the authors proposed a crack arresting strategy that is essentially based on adherends notching (Figure 1f). In particular, the effect of a single notch located at one of the mating surfaces was evaluated using the virtual crack closure technique (VCCT). The results indicated that because of the notch, a significant portion of the strain energy supplied through the external work was used to deform the adherends rather than crack growth. As a result, crack propagation was delayed, and the apparent toughness was significantly enhanced. Therefore, while prior studies have focused on joint strength, the literature review demonstrated that there is much additional scope in using adherends notching to control the mechanics of crack propagation in adhesive joints. However, to date, this topic has received limited attention and more research should be conducted to understand the interplay between notches geometry and architecture (i.e., number of notches, spacing, and depth) and crack propagation/arrest.

The aim of this study was to complement existing research and provide a contribution toward filling the above gaps. In particular, we sought to elucidate the mechanics of crack propagation in bonded joints featuring an array of notches perpendicular to crack growth direction. A schematic of the intended geometry is provided in Figure 1g. The focus was placed on a configuration that can be possibly obtained using standard manufacturing methods, such as pulsed laser micro-machining and cutting [22,23]. Previous papers emphasised that the notches geometry plays an important role in joint strength. Likewise, here, we propose a parametric investigation using a cohesive zone approach to understand the effect of notch architecture (e.g., spacing, depth, etc.) on selected performance metrics, such as the load–displacement response, the evolution of crack length, and the breakdown of dissipated energy. The outcome of the notching strategy on joint strength was found to be dependent on the nature of the structural adhesive. Likewise, we expected that bondline properties, i.e., brittle versus ductile adhesives, would also affect the toughening effect induced by the notches. Recall that fracture of ductile adhesives is accompanied by a considerable process zone extending ahead of the crack tip. Recent investigations on peeling of elastic and elasto-plastic adherends with variable bending stiffness, or interfacial adhesion, showed that an extended fracture process zone exerts a smoothing effect on the enhancement of peel strength [24,25,26]. Therefore, we varied the cohesive properties to manipulate the process zone size and ascertain the corresponding effect on crack growth.

The manuscript is organised as follows: Section 2 describes the FE model, the corresponding inputs, and details of the extracted performance metrics. Section 3 summarises the obtained results and highlights the effect of notch geometry and cohesive properties on the selected performance metrics. Finally, Section 4 provides conclusions and recommendations for follow-up research.

## 2. Model Description

### 2.1. DCB with Notched Adherends

**Figure 2:**
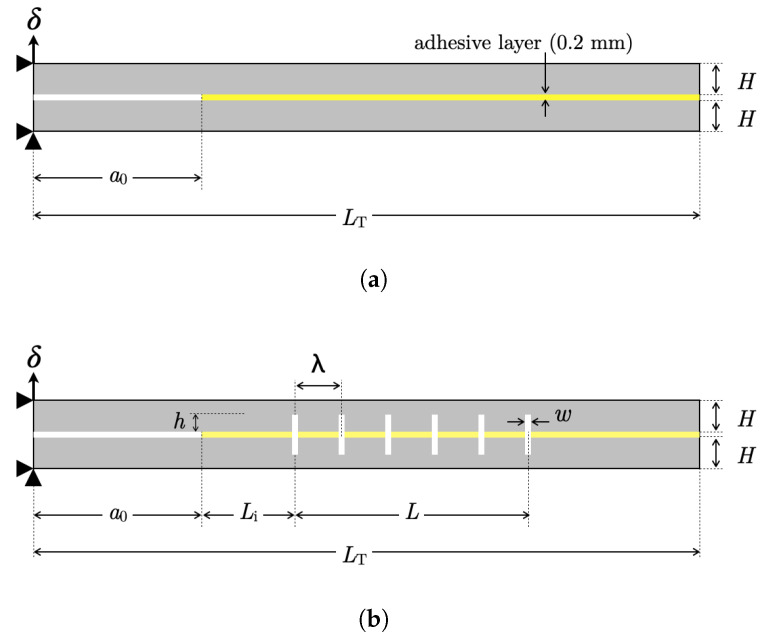
Schematic of the (**a**) baseline and (**b**) notched DCB joints.

A double cantilever beam (DCB) comprising two beams adjoined by an epoxy adhesive layer was considered as a model material system to study crack propagation under mode I loading. The baseline DCB is shown in Figure 2a. The initial crack length is $a_{0}$ = 40 mm, the total length of the DCB is denoted as $L_{T}$ = 140 mm, and *H* = 6 mm is the height of each adherend; the width *B* = 15 mm. The thickness of the adhesive layer is assumed to be 0.2 mm. These values were kept constant throughout all simulations. Finally, δ represents a concentrated (nodal) upward displacement whose value was adjusted throughout the simulations to achieve comparable crack growth across the various models. Notice that the schematics reported in Figure 2 include the boundary conditions employed in the FE simulations. In particular, the symbols ▸ and ▴ denote constrained nodal point displacements in the horizontal and vertical directions, respectively; ↑ represents the applied nodal point (opening) displacement.

A similar DCB model with notches was prepared, and a schematic depiction showing the relevant geometrical features is provided in Figure 2b, where λ is the pitch/spacing of the notches, with *w* and *h* being the width and height, respectively. The total number of notches is denoted as *p*, with the first one located at a distance $L_{i}$ from the initial crack tip and the remaining ones distributed over a distance *L*. The FE model of the notched DCB involved the following constant geometrical properties: L≈ 60 mm and $L_{i}$ = 20 mm. As shown below, *w* and *h* were varied throughout the analyses. Likewise, the number of notches p≈1+L/λ were also adjusted as λ was changed to establish the effect of notch spacing on crack growth.

### 2.2. Finite Element Modeling Details

**Figure 3:**
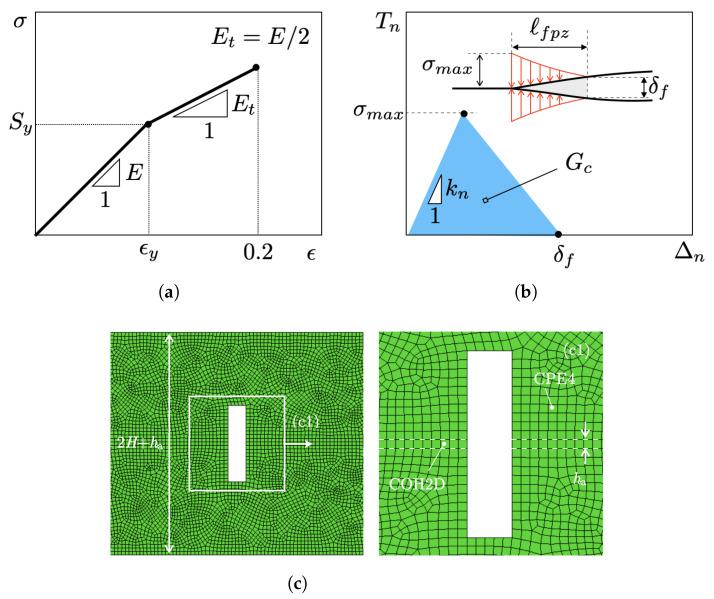
(**a**) Schematic stress–strain (σ−ϵ) diagram; (**b**) bilinear traction–separation relation employed in FE simulations of crack growth - the insert highlights cohesive tractions acting across the crack faces within the fracture process zone; (**c**) details of an exemplary FE mesh (w/H = 1/6). (**c1**) Close-up view of the FE mesh around a notch.

A FE model of the DCB was developed with ABAQUS/Standard. Material properties were selected taking inspiration from our previous study focused on bioinspired polyamide/epoxy joints [13]. A schematic of the σ−ϵ diagram is provided in Figure 3a. The substrates featured a linear elastic isotropic behaviour up to the yield point, followed by linear hardening. The input properties were as follows: Young’s modulus *E* = 1650 MPa, Poisson’s ratio ν = 0.4, yield stress $S_{y}$ = 31.4 MPa, $E_{t}=E/2$ ($E_{t}$ is the tangential modulus), and ultimate strength $S_{ut}$ = 45 MPa.

The actual crack growth process within the adhesive layer is accompanied by the formation of a fracture process zone (fpz) featuring damage mechanisms such as microcracking, crazing, and plasticity [7]. In this study, a simplified model was considered, whereas the adhesive layer was replaced by a single row of cohesive elements featuring a bilinear traction-separation relation linking the normal traction ($T_{n}$) and the conjugate opening displacement ($\Delta_{n}$) [27]. As such, the damage occurring within the fpz was lumped into the crack line and modelled with the traction-separation relation. A similar modelling approach has been also used to analyse fractures in composites [28] and granular nanomaterials [29]. Schematics of the cohesive model and fpz are shown in Figure 3b, which highlights the main controlling input variables: cohesive energy ($G_{c}$), cohesive strength ($\sigma_{max}$), initial stiffness ($k_{n}$), and final opening displacement ($\delta_{f}=2G_{c}/\sigma_{max}$) corresponding to $T_{n}$ = 0. It should be noted that $\ell_{fpz}$ is the distance spanning from the cohesive crack tip to the point where $T_{n}$ = $\sigma_{max}$. The following baseline values were used in this study: $G_{c}$ = 0.4 N/mm, $k_{n}$ = 8500 MPa/mm, and $\sigma_{max}$ = 10 MPa. The shape of the cohesive model was not included in the investigation as we assumed the size of the cracked specimen to be larger than that of fpz ($\ell_{fpz}$) [30].

The FE model was built in ABAQUS/Standard. Continuum bilinear plane strain quadrilateral elements with four nodes and full integration (CPE4) were used for the substrates. The adhesive layer was replaced by a single row of cohesive elements, each having four nodes and two integration points (COH2D). The benchmark DCB comprised 22,175 CPE4 and 550 COH2D, for a total of 22,925 nodes. The notched DCB model with the most refined mesh comprised 51,956 CPE4 and 550 COH2D, for a total of 53,628 nodes. The mesh was generated using the advancing front algorithm and the free mesh technique available in ABAQUS/CAE. An exemplary image of the FE mesh made around a notch, including a close-up view, is shown in Figure 3c. Using such approach, the size of the smallest CPE4 element was about 0.1 mm, while the size of the smallest cohesive element was 0.2 mm. The chosen mesh details allowed for three to five cohesive elements to be always present within the process zone, thus enabling an accurate representation of the tractions ahead of the crack tip [31]. In addition, one to five CPE elements were always included to model the width of the notches, and more than five were always placed along their depth. With the above mesh details, a reasonable balance between accuracy and computational effort was achieved.

In our displacement-controlled simulations, viscous regularisation was needed to overcome the instabilities associated with crack growth past a notched region. In actual experiments, the viscous energy might well correspond to the kinetic energy or the elastic wave radiation occurring in the course of unstable crack growth [24]. The viscosity affects the FE results, i.e., it leads to increased cohesive tractions, thereby introducing a certain degree of fictitious energy dissipation to reach equilibrium. For this reason, in FEA, the actual value of viscous energy is approached using progressive smaller values of the viscous coefficient (μ), such that below a certain μ, there is no difference between FE predictions [32]. In this study, this value was found using a trial-and-error approach and was equal to μ = 1 × 10${}^{-6}$ s. This approach was successfully employed in our previous study to analyse the snap-through cracking process occurring in bioinspired adhesive joints [13].

### 2.3. Performance Metrics

To assess the effect of notches’ geometry and arrangement as well as the influence of cohesive properties, we investigated the following performance metrics: load–displacement traces, crack size versus applied displacement plots, and the effective fracture energy ($G_{eff}$). This last was defined as the total energy per unit crack area involved through the fracture process that comprised the plastic energy ($W_{p}$) involved in the crack propagation process.

Any of the above performance metrics (Ψ) can be affected by a few variables, including cohesive and bulk (adherends) material properties as well as the geometry and arrangement of the notches:

(1) $$\Psi =f(\underset{\mathrm{cohesive properties}}{\underset{︸}{G_{c},\sigma_{max}}},\underset{\mathrm{bulk material properties}}{\underset{︸}{E,\nu ,S_{y},E_{t}}}\;,\underset{\mathrm{notches}’\mathrm{geometry}}{\underset{︸}{H,h,\lambda ,L,w}}\;).$$

The above equation is recast in a nondimensional form with the following dimensionless invariants:

(2) $$\bar{\Psi }=g(\frac{E}{S_{y}},\frac{E_{t}}{E},\underset{\mathrm{variables included in the analysis}}{\underset{︸}{\frac{\sigma_{max}}{S_{y}},\frac{G_{c}E}{\sigma_{max}^{2}H},\frac{w}{H},\frac{h}{H},\frac{\lambda }{H}}}\;)$$

The first two nondimensional groups were set constant throughout the simulations using the inputs previously shown. The remaining variables were varied in order to monitor the resulting mechanical response and energy dissipation. In particular, the following values were considered: $\sigma_{max}$/$S_{y}$ = 1/6–4/3; $\xi =G_{c}E/\sigma_{max}^{2}H$ = 0.5–5.0; w/H = 1/40–1/6; h/H = 1/3–2/3; λ/H = 5/6–10/3. The variables $\sigma_{max}/S_{y}$ and ξ play an important role in the outcome of the FE simulations. The former affects the plastic energy involved in deformation and fracture of the joints, as it increases with $\sigma_{max}$. The latter is a nondimensional quantity that scales with the fracture process zone length ($\ell_{fpz}$) [33]:

(3) $$\ell_{fpz}=\alpha \frac{G_{c}E}{\sigma_{max}^{2}}$$

and where α is a constant typically in the range of 0.2 ≤α≤ 1 [31]. It is apparent that $\ell_{fpz}$ largely varies with $\sigma_{max}$, e.g., a 10-fold variation in cohesive strength produces a 100-fold decrease. Previous studies by one of the authors and others have shown that $\ell_{fpz}$ can play an important role in crack propagation in heterogeneous materials [24], as well as in the peeling strength of adherends with varying bending stiffness [25] or interfacial adhesion [26]. Therefore, the effect of $\ell_{fpz}$ was included in the subsequent FE simulations.

## 3. Results and Discussion

### 3.1. Mechanics of Crack Growth (Elastic Analysis)

**Figure 4:**
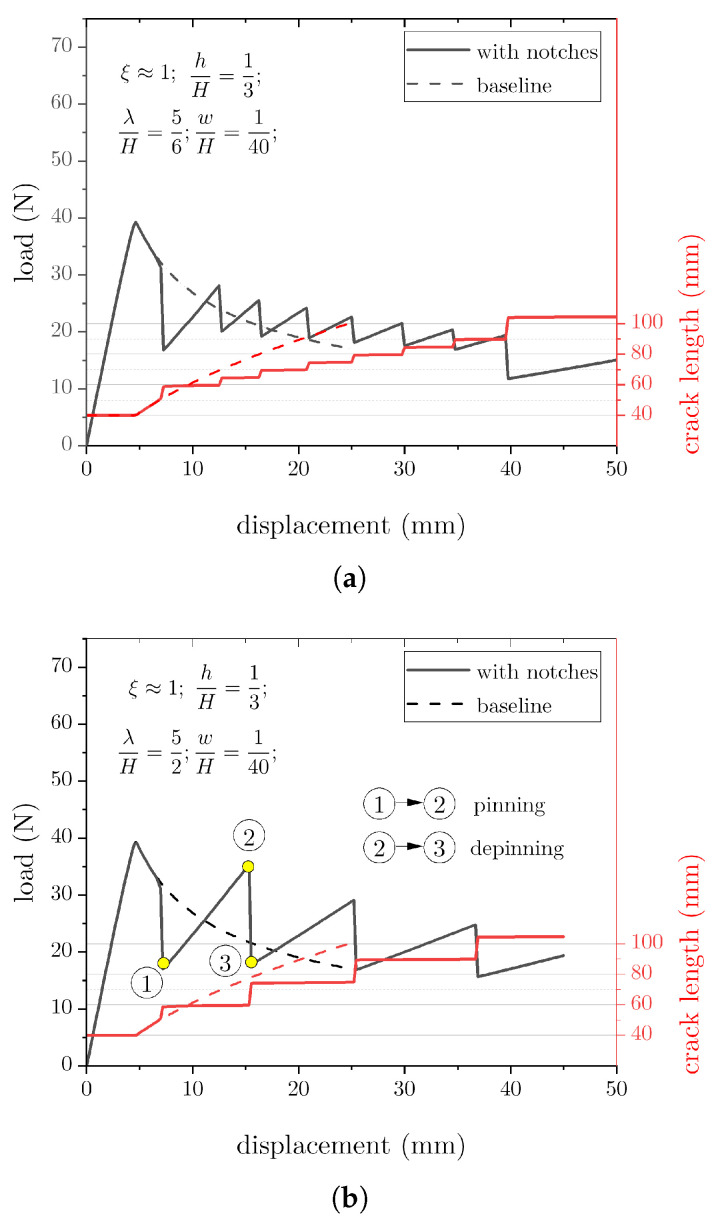
Load–displacement and crack length data for DCB adhesive joints with varying notch spacing. (**a**) λ/H=5/6; (**b**) λ/H=5/2.

We begin the analysis by showing the load–displacement responses of the joints with multiple notches (w/H = 1/40) that are spaced evenly at a distance λ/H varying between 5/6 and 5/2. The load–displacement traces of baseline and notched DCBs are provided in Figure 4 along with the corresponding crack length. Notice that these simulations assume a linear elastic isotropic behaviour for the adherends (no plasticity). The results demonstrate the occurrence of an unstable pinning/depinning process. In particular, the initial response of notched joints overlaps with the baseline because the distance between the first notch and the crack tip was large enough to prevent any perturbation. After that, the load–displacement traces exhibit a series of local maxima as the crack snaps in dynamic fashion through the notches. At each transition, the load increases, reaches a peak, and suddenly drops to a lower level, while the external displacement does not change. As expected, λ/H = 5/6 consistently displayed the most load fluctuations with smaller peak loads. However, as shown in Figure 4b, the magnitude of load fluctuations can be tuned by increasing the spacing λ.

To better understand the pinning/depinning process, we now focus on the portion of the load–displacement curve highlighted by the points ➀, ➁ and ➂ in Figure 4b. As the load increases, i.e., ➀→➁, there is no significant variation in crack size. However, once the load reaches the peak ➁, any further increase in applied displacement induces a sudden crack jump and a load drop ➁→➂. Therefore, the notches delay crack propagation and enable an increased applied loading and work of fracture (i.e., area below the load–displacement curve).

**Figure 5:**
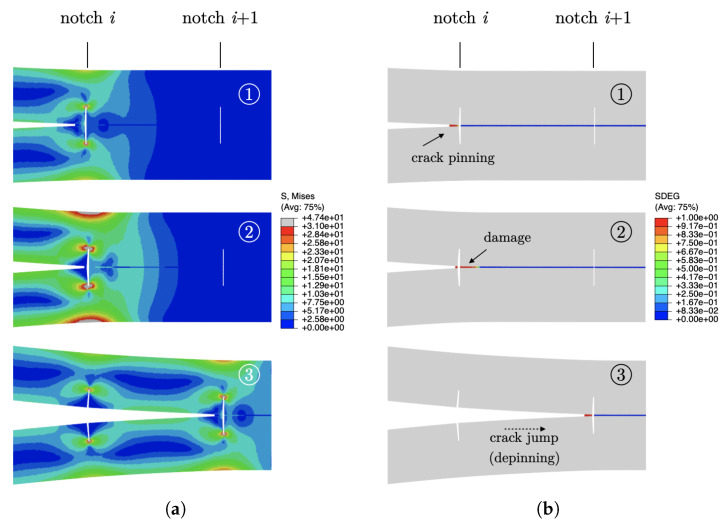
FE snapshots of the crack propagation process occurring at the load–displacement points highlighted in Figure 4b. Fringe plots and ensuing colour bars that illustrate (**a**) the von Mises stress and (**b**) the scalar stiffness degradation (SDEG).

FE snapshots of the deformed mesh were extracted at previous points ➀–➂ and are provided in Figure 5. The snapshots include fringe plots of the von Mises stress and the scalar stiffness degradation (SDEG). This last captures the state of damage within cohesive elements and assumes values between zero (no damage) and one (complete failure). At point ➀, the bondline between two consecutive notches is still not fractured. While damage within cohesive elements next to the notch accrues, the applied load and the von Mises stress build up and reach a peak (➁). At this stage, the strain energy supplied through the external work is stored in the adherends and does not contribute to crack growth. As a consequence, the propagation process is delayed, and the joint is able to withstand a higher external force before macroscopic crack propagation occurs. Following an additional increase in the applied displacement, a sudden load drop is observed because of the unstable crack extension, and the crack front reaches the next notch (➂). After that, the process restarts and repeats itself in a substantially identical manner.

### 3.2. Energy Breakdown

As illustrated in Figure 4, for a given crack increase Δa = 100 mm, the addition of notches leads to an increased global work of fracture, i.e., about 40% (λ/H=5/2). The previously described unstable pinning/depinning process is deemed responsible for such enhancement. In analogy with stick–slip phenomena in tribology, the excess energy can be mechanically dissipated through stress wave propagation, kinetic energy, and other sources such as heat and vibrations.

**Figure 6:**
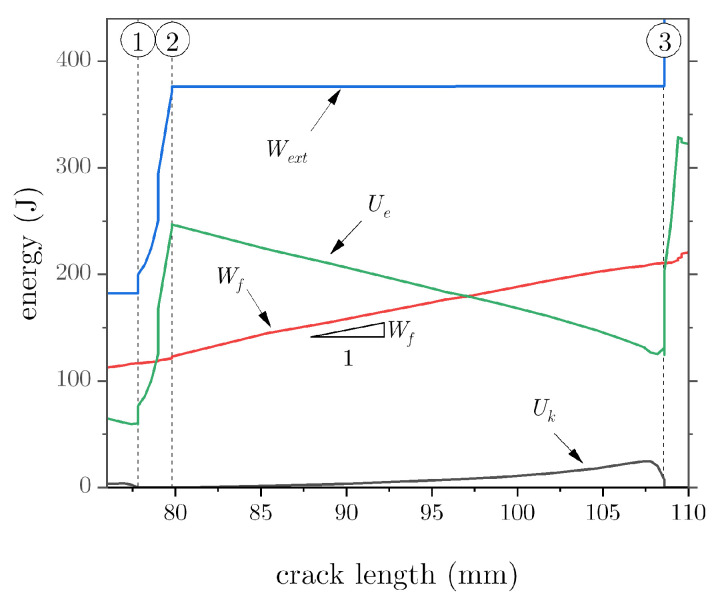
Energy breakdown as a function of crack length.

To further decipher the energy budget involved in the crack propagation process, we conducted a dynamic implicit analysis with a slow displacement rate (0.5 mm/min) to simulate quasistatic conditions. The obtained results are depicted in Figure 6, which comprises the total external work ($W_{ext}$), the strain energy ($U_{e}$), the fracture energy ($W_{f}$), and the kinetic energy ($U_{k}$). These variables were obtained as part of the ABAQUS history output, with identifiers ALLWK, ALLSE, ALLDMD, and ALLKE, respectively.

The results illustrate a steep increase in the external work during the quasistatic or pinning phase ➀–➁, which is mostly converted into strain energy. However, once the crack tip breaks through the notch, the strain energy decreases, while the fracture energy linearly increases. In the process, the external work is essentially constant. This is the dynamic crack growth regime ➁–➂, whereby the strain energy undergoes a fast decrease that cannot be balanced by the fracture energy and, as a result, kinetic energy appears. It should be noted from Figure 6 that the rate of change in the fracture energy as a function of crack length is approximately constant, i.e.,

(4) $$\frac{\partial W_{f}}{\partial a}=\mathrm{constant}.$$

Because a=a(t), it follows:

(5) $$\frac{\partial W_{f}}{\partial a}=\frac{\partial W_{f}}{\partial t}\frac{\partial t}{\partial a}=\frac{1}{\dot{a}}\frac{\partial W_{f}}{\partial t}.$$

As observed earlier, during the quasistatic crack growth phase ➀–➁, crack propagation is delayed, i.e., $\dot{a}$ decreases. It follows that Equation (4) holds only if the rate of dissipation ($\partial W_{f}/\partial t$) decreases too, implying that the supplied external work is converted into strain energy and, thus, it is not available for crack growth.

### 3.3. Apparent Fracture Toughness

The pinning/depinning process observed herein was also reported in previous related studies and is broadly considered to be a toughening mechanism. It results from the interaction between the crack front and a local heterogeneity, such as elastic stiffness [24] or thickness of the adjoined layers [25], as well as patterned interfacial adhesion [26]. In the present study, similarly to the study of Kanani et al. [19], the notches split the bondline in smaller sections. As such, crack propagation is interrupted whenever the crack front meets a notch, which represents the heterogeneity giving rise to the pinning/depinning process. Because of the unstable growth, the apparent fracture toughness of the joint can significantly increase. To show this point, we analysed the apparent toughness ($\hat{G}$) determined as follows:

(6) $$\hat{G}=\frac{1}{B}\frac{d}{da}\left[\int_{0}^{\bar{\delta }}P\left(\delta \right)d\delta -\frac{P\bar{\delta }}{2}\right].$$

**Figure 7:**
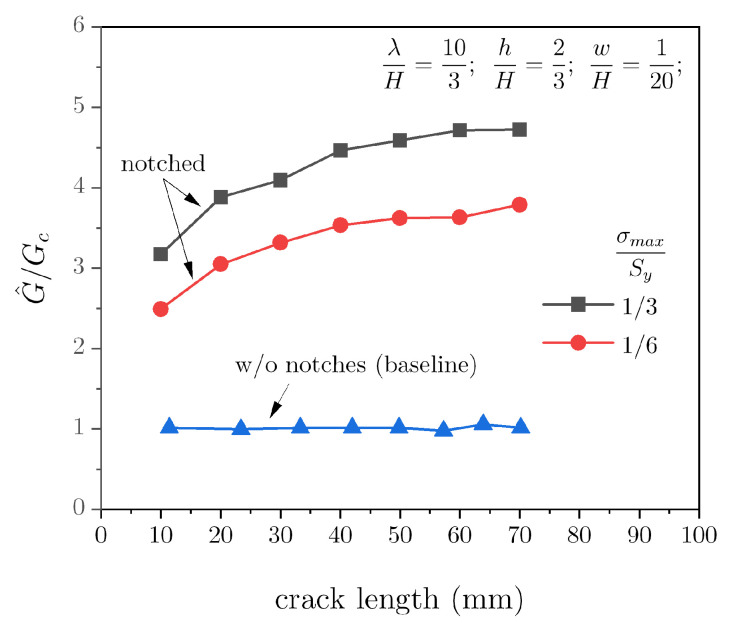
Apparent fracture toughness of DCB joints with notched adherends.

The first term within parentheses represents the total work supplied to the system, and the second one is the corresponding portion stored as elastic energy. As in previous analyses, the above equation is based on the assumption of global elastic deformations. However, it is still quite instructive, as it allows pinpointing the effect of adherends notching on crack growth resistance. The obtained results are reported in Figure 7 after normalisation with respect to the input fracture toughness. For these simulations, we employed deeper notches and lower cohesive strengths to emphasise that the mechanical behaviour can be tuned by combining appropriately material and geometrical properties.

Notice that we used a similar approach to extract the toughness of the baseline joint and, as expected, the results are close to unity after normalisation (except for small integration errors). For the notched DCB joints, the fracture resistance increases with the crack length because the stretchability of the adherends and the energy absorbed and expelled during the pinning/depinning process increase accordingly. This is further demonstrated by noting that an adhesive layer with a higher cohesive strength further increases the apparent toughness.

### 3.4. Effect of Substrates Plasticity

#### 3.4.1. Analysis of Applied Load and Crack Growth as a Function of the Opening Displacement

**Figure 8:**
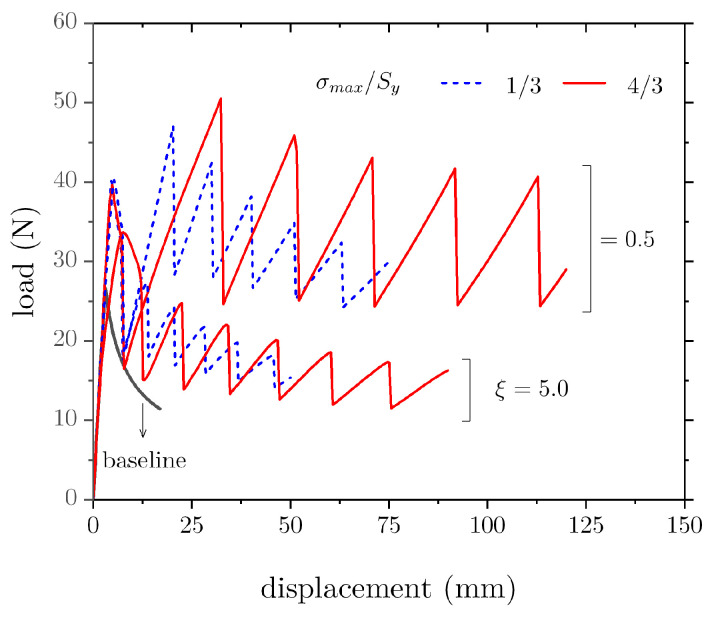
Effect of cohesive properties on the load–displacement response of a notched DCB adhesive joint.

We now assess the effect of bondline properties (ξ and $\sigma_{max}/S_{y}$) on the load–displacement response of the DCB assuming an elastoplastic behaviour. For these simulations, h/H = 1/3, λ/H=1.5, and w/H = 1/40. The results are shown in Figure 8 for an approximate crack growth of about Δa = 60 mm. The overall applied displacement significantly increased. Indeed, while the baseline DCB requires an applied displacement of 25 mm to achieve the above stated crack growth, for the notched specimens the opening needed increases to about 120 mm. For a given ξ, the applied displacement also increases with $\sigma_{max}/S_{y}$. It should be noted that the increased stretchability is a characteristic of the adherends notching technique, but it is further promoted through the elastoplastic material behaviour. In the experiments by Sankatar and Simmons [15], the enhancement in SLJ strength was accompanied by a three-fold increase in joint deformation. The authors considered it to be an improvement because the notches facilitated the plastic deformation of the adherends before failure. The applied loading dramatically increases as ξ decreases. While a similar outcome is observed as $\sigma_{max}/S_{y}$ increases, the effect of ξ is much stronger on both the peak loads and the ensuing work of separation.

We note in passing that an order of magnitude decrease in ξ caused significant decreases in applied load and absorbed energy needed to sever the joint. However, unlike previous related studies [24,25,26], despite the substantial increase in ξ (or the fracture process zone size), there is still a significant improvement in the mechanical behaviour with respect to the baseline.

**Figure 9:**
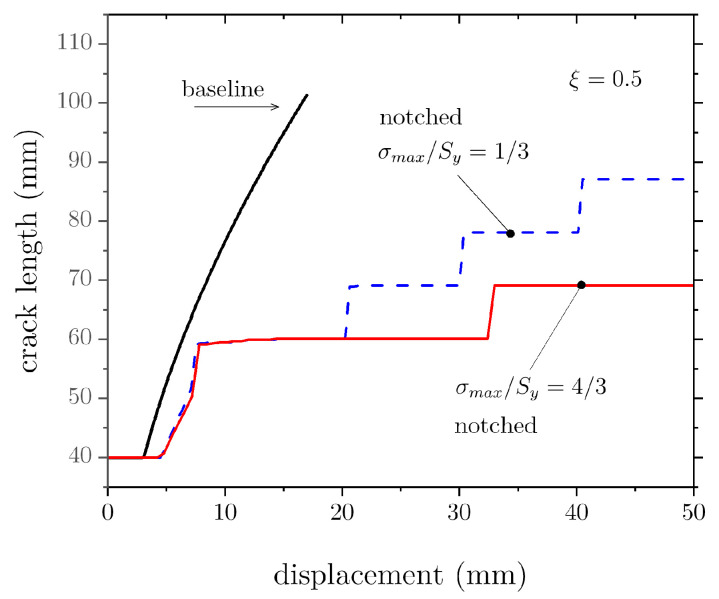
Effect of cohesive strength on the crack length versus displacement plots before and after the addition of notches.

As discussed earlier, notched adherends can delay crack propagation with respect to the baseline joint. This point is best illustrated by analysing the crack length versus applied displacement, as shown in Figure 9, which demonstrates a significant difference between baseline and notched DCBs. In particular, crack growth is limited in the notched specimens, and it further reduces as $\sigma_{max}/S_{y}$ increases. The delayed crack growth occurs because most external work is converted into adherends elastic (and plastic) deformation rather than being used for fracture of the bondline.

#### 3.4.2. Effect of Notch Spacing and Geometry

**Figure 10:**
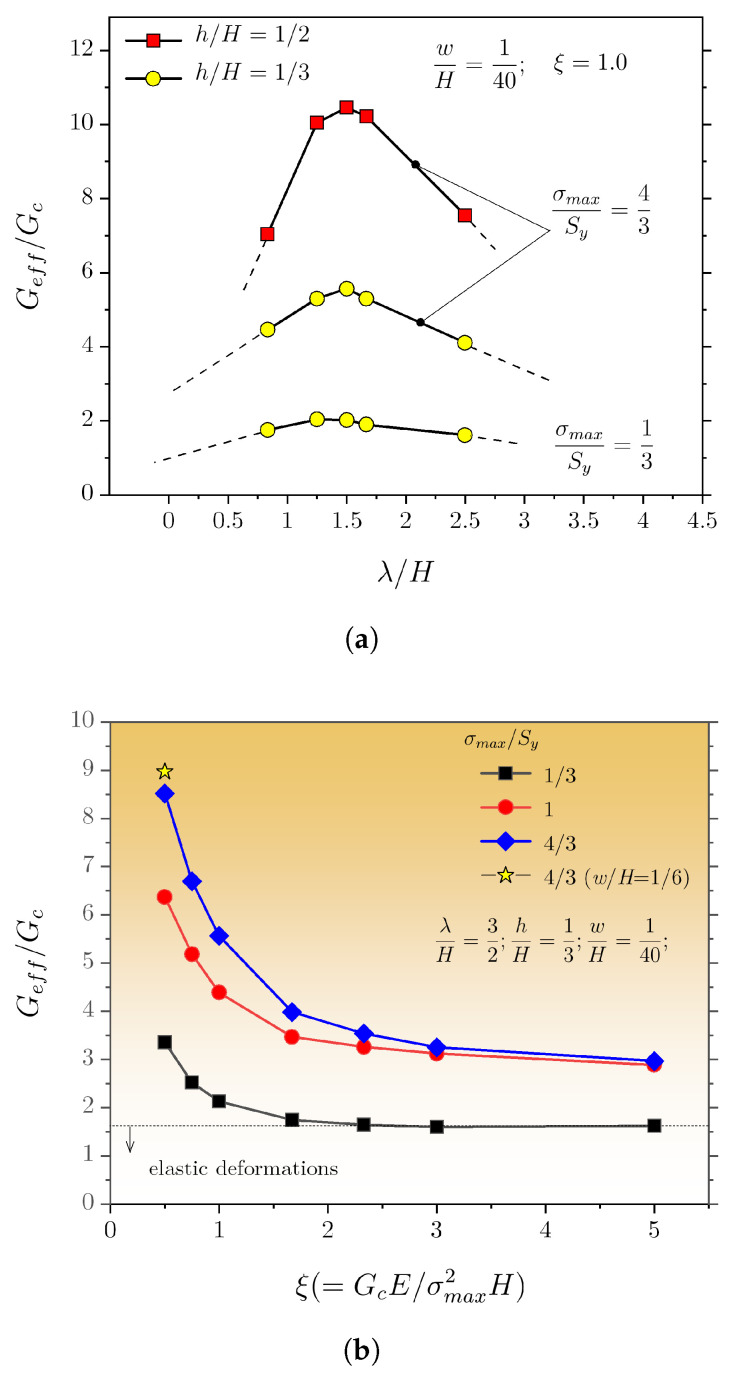
Evolution of the effective dissipated energy as a function of (**a**) λ/H and (**b**) $G_{c}E/\sigma^{2}H$.

We now turn our attention to the existing interplay between notch architecture, i.e., spacing (λ) and depth (*h*), and the bondline properties. The notch width (*w*) was kept constant in these simulations. The results are provided in Figure 10a and show a strong variation in $G_{eff}$ with the spacing. For larger spacing, the effective energy decreases, as in the limit of infinite spacing, the mechanical behaviour of a baseline DCB joint would be attained. A peak value occurs at approximately λ/H = 3/2, but the amplitude proportionally scales to $\sigma_{max}/S_{y}$. This is readily explained by noting that the main contributor to the enhanced energy dissipation is the bulk plasticity associated with the deformation of the DCB adherends. If plasticity is suppressed, the enhancement in $G_{eff}$ with the spacing is much reduced (see $\sigma_{max}/S_{y}$ = 1/3) but still significant compared with the baseline configuration. Additionally, an increase in notch depth of about 1 mm induces a more than two-fold increase in the peak value of $G_{eff}$. This shows that the effect of notches geometry is as much as important as the bondline material properties.

For a given spacing, i.e., λ/H = 3/2, we assessed the variation in $G_{eff}$ with ξ. The results are provided in Figure 10b and show that the energy needed to sever the joints increases rather steeply as ξ decreases, especially for increasing $\sigma_{max}/S_{y}$. Furthermore, w/H was extended from 1/40 to 1/6 to ascertain the effect on dissipated energy (the remaining variables were not changed). As shown in Figure 10b, a much increased notch width still provided a significant increase of dissipated energy. This is an important outcome, as using a larger notch may be of more practical experimental value.

**Figure 11:**
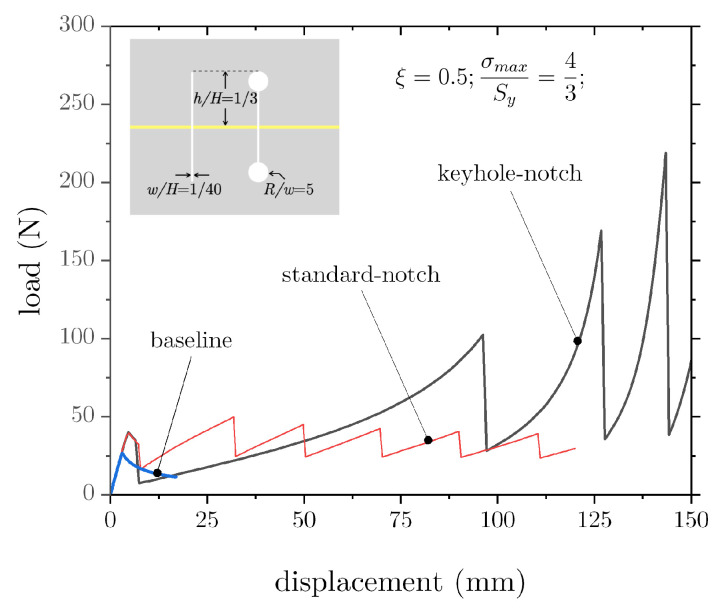
Effect of notch geometry on the load–displacement response of DCB joints.

The above analysis suggests enhanced sensitivity to the notch geometry. For this reason, we explored the effect of an array of keyhole-like notches that still had the same depth as the previous ones (h/H = 1/3) but featured a rounded end, such that R/w = 5. The obtained load–displacement curves and a schematic of the notch are shown in Figure 11. The results further demonstrate that tailoring the architecture of the notches can have an outstanding impact on joint performance.

## 4. Closing Remarks and Conclusions

Previous studies by us and others have shown that the strength and toughness of adhesive joints can be tailored by controlling the architecture of the adjoined layers. In this study, we found that a simple modification of the adherends, consisting of an array of equally spaced notches (machined in the direction orthogonal to the crack growth direction), can have a profound effect on the mechanics of crack propagation. The notches add compliance to the system and increase the total energy required before separation of the joints. In particular, an unstable pinning/depinning mechanism delays the crack propagation process. While adherends plasticity can be a major contributor to the enhanced dissipation, similar to what was reported in related studies [24], we showed that the associated snap-through cracking involves additional sources of dissipation, i.e., kinetic energy. Additionally, the geometry of the notches, including depth and spacing, plays a crucial role and allows for tunable mechanical behaviour. For instance, notches with a rounded end (within the adherends) can largely increase the overall dissipation (≈×4).

As a limitation of the current study, it is noted that the conceived finite element model only allows damage and failure via detachment of the joined adherends. In other words, we accounted for the effect of adherends plasticity, but we surmised the absence of substrate fracture. This simplified model can represent strong adherends bonded via a tough epoxy or by a diffusion bonding process. Accounting for failure within the adjoined layers would expand the scope of the present study. For example, the application of refined regularised models (e.g., phase-field models) may represent a valuable tool for the analysis of complex cracking patterns in arbitrary geometries and dissimilar materials [34,35].

Finally, we did not explore the effect of notch width beyond w/H = 1/6. While this would be a useful extension of this study, we highlight that the main interest herein was to establish the effect of notches that do not significantly change the joint geometry/thickness. Future research should include experimental testing to further assess and refine the methodology.

## Data Availability

The data reported in this study are available upon request.

## Abbreviations

The following abbreviations are used in this manuscript:

FE	finite element
SLJ	single lap joint
DCB	double cantilever beam
VCCT	virtual crack closure technique
CZM	cohesive zone model
*B*	width of the specimen
*H*	thickness of the adherend
$h_{a}$	thickness of the adhesive layer
$L_{T}$	total length of the specimen
*w*	notch width
*h*	notch depth
λ	notch spacing
$L_{i}$	notch-free portion of the bondline (from initial crack tip)
*L*	notched portion of the bondline
*p*	number of notches
$a_{0}$	initial crack length
Δa	crack increment
$\dot{a}$	crack speed
*E*	Young’s modulus
ν	Poisson’s ratio
$S_{y}$	yield strength
$E_{t}$	tangential modulus
$S_{ut}$	ultimate strength
fpz	fracture process zone
$\ell_{fpz}$	length of fpz
$T_{n}$	opening traction in cohesive zone
$\Delta_{n}$	conjugated opening displacement
$k_{n}$	initial stiffness of the cohesive model
$G_{c}$	cohesive fracture energy
$\sigma_{max}$	cohesive strength
$\delta_{f}$	final opening displacement
μ	viscous coefficient
$G_{eff}$	effective fracture energy
$\hat{G}$	apparent toughness
$W_{ext}$	external work
$W_{f}$	fracture energy
$U_{e}$	strain energy
$U_{k}$	kinetic energy
Ψ	performance metric
